# Synergistic insecticidal effects of zinc-loaded zeolite nanoparticles combined with essential oils against *Callosobruchus maculatus*

**DOI:** 10.1038/s41598-025-15752-9

**Published:** 2025-08-28

**Authors:** Ahmed Mohamed El-Bakry, Hanan Farouk Youssef, Nasr Mohamed Abdelmaksoud, Nahed Fawzy Abdel-Aziz, Elham Ahmed Sammour

**Affiliations:** 1 Pests and Plant Protection Department, Agricultural and Biological Research Institute, National Research Centre, Dokki, Cairo Egypt; 2 Ceramics, Refractories and Building Materials Department, Advanced Materials Technology and Natural Resources Institute, National Research Centre, Dokki, Cairo Egypt

**Keywords:** Zeolite-A, Zeolite-X, Inert dusts, *Rosmarinus officinalis*, *Pimpinella anisum*, Co-toxicity factor, Mass spectrometry, Plant sciences, Biological techniques, Nanobiotechnology, Nanoparticles

## Abstract

*Callosobruchus maculatus* (F.) is a serious pest that causes post-harvest losses, which is a threat to global food security, therefore there is need to develop sustainable pest management strategies. This study investigates the synergistic insecticidal effects of zinc-loaded zeolite nanoparticles in combination with essential oils from *Rosmarinus officinalis* (L.) and *Pimpinella anisum* (L.) against *C. maculatus* adults and their progeny. Zeolite-A and zeolite-X were synthesized and characterized by X-ray diffraction (XRD), scanning electron microscopy (SEM) and energy dispersive X-ray spectroscopy (EDS), and found to be highly crystalline and successfully zinc functionalized. The chemical profiles of the essential oils were elucidated by gas chromatography-mass spectrometry (GC-MS). The results showed that zeolites alone had moderate insecticidal activity against the tested insect. Zeolites loaded with zinc enhanced insecticidal activity on *C. maculatus*. Combining zeolites with essential oils further increases insecticidal activity, with LC_50_ values ranging from 161 to 306 mg/kg. Zeolite nanoparticles and *P. anisum* essential oil formulation was the most effective in killing *C. maculatus* adults and progeny. Co-toxicity factor analysis indicated that there were synergistic effects between the essential oils and zeolites, especially between *P. anisum* and Zn-zeolite-A. Morphological examination of treated *C. maculatus* adults via SEM revealed cuticle abrasions, desiccation areas, and damage to sensilla, indicating a physical mode of action for the zeolites. This study suggests that zeolite nanoparticles and essential oil combinations can be used as eco-friendly insecticides for the management of *C. maculatus* in stored cowpea seeds.

## Introduction

Grain legumes are crucial in providing nutrition to both humans and animals across the globe^1^. Cowpeas, *Vigna unguiculata* (L.) (Fabales: Fabaceae), stand out as an essential grain legume, offering a rich energy source, protein, vitamins, minerals, and dietary fibre. Sprouting legumes improves nutritional absorption and digestion, making them significant in human nutrition^2^. Incorporating whole-grain cereals and grain legumes can act as a meat alternative in terms of being a protein, zinc, and iron source. Furthermore, this combination can enhance dietary fibre and folate intake, which are often inadequately consumed^3^. Cowpeas are an essential crop for food security but are vulnerable to insect damage^4^. Infestations by *Callosobruchus maculatus* (F.) (Coleoptera: Chrysomelidae) can lead to post-harvest losses that threaten sustainable food security and cause significant economic harm to farmers worldwide. This pest damages stored seeds, resulting in reduced nutritional quality, decreased seed viability, dry weight loss, and diminished market value^5^.

The predominant approach for managing cowpea bruchid in storage places is through the use of fumigants such as phosphine and other chemical insecticides. Although synthetic insecticides are considered effective in controlling insect pests, their long-term environmental impact, leftover toxicity in food, and the development of insect resistance have necessitated the development of safer alternatives^6^. Additionally, these insecticides can be hazardous to users^7^. Consequently, there has been a shift toward exploring natural products as substitutes for traditional insecticides^8,9^. Research has focused on the potential of plant essential oils and inert dust as viable options for controlling pests instead of relying on chemical control methods^10^. Plant-derived essential oils are organically produced secondary metabolites currently employed in various applications, including fragrance, cosmetics, herbal medicine, and nutrition.

These oils can also serve as eco-friendly insecticides^11^. Various studies have concentrated on utilizing essential oils extracted from plants to manage insect pests in stored grains^12–14^. *R. officinalis* (L.) (Lamiales: Lamiaceae) and *P. anisum* (L.) (Apiales: Apiaceae) essential oils have been found to be effective against stored product insects as a repellent, fumigant, and contact insecticides due to their active ingredients like 1,8-cineole, camphor, and anethole^15,16^. However, plant essential oils have the potential to leave a long-lasting scent. Their high concentration could cause food to possess an overpowering aroma and unpleasant taste^17^. However, when essential oils are used in low quantities, the protective impact may be lessened since certain active elements may move into the environment. This poses a challenge when using botanical essential oils as grain treatments, as their efficacy against insects needs to be increased while decreasing residues in food commodities^18^.

Inert dusts are utilized to manage insects in stored grain. Unlike conventional contact insecticides, inert dusts work through their physical characteristics, which often results in a slower response time^19,20^. Zeolites are microporous, crystalline aluminosilicates that can be found naturally or synthesized^21^. They have a wide range of applications in agriculture, including improving soil properties, promoting plant growth, increasing metal content in plant aerial parts, serving as food additives for domestic animals, and acting as carriers for fertilizers^22^. Zeolites can effectively control insect populations by abrading or adsorbing the epicuticular lipids on their bodies. This causes the insects to lose water and eventually die from dehydration^23^. Hard, non-sorptive particles are responsible for the abrasion of epicuticular lipids, while sorptive particles are responsible for their adsorption^6^. Zeolites are generally considered safe for the environment, animals, and humans. They are listed as safe for human consumption by the FDA and are classified as non-toxic by the World Health Organization’s International Agency for Research on Cancer^24,25^. Like other inert dusts, zeolites take some time to cause insect mortality. Moreover, high application rates of zeolites can alter stored grains’ bulk density and physical properties^22^. One potential solution to this issue is combining essential oils with low concentrations of zeolites. Zinc is one of the most commonly used ions to be mixed with zeolite structure to produce biological activity, owing to its excellent stability and the broad spectrum of efficiency against numerous types of bacteria, viruses, germs, and fungi^26^.

In this study, zeolites were combined with essential oils as a novel approach to gain a rapid action of inert dusts against stored product insects. Also, the study aimed to mitigate odors caused by high concentrations of essential oils. Through comprehensive experimentation and analysis, this research aimed to evaluate the cumulative insecticidal mortality of two types of zeolites (zeolite-A and zeolite-X) and their zinc-loaded zeolites, as well as their formulations with *R. officinalis* and *P. anisum* essential oils, against the adults of *C. maculatus* and their progeny production.

## Materials and methods

### Plant materials and essential oil extraction

Rosemary leaves, *R. officinalis* and aerial parts of anise, *P. anisum* were collected from the medicinal and aromatic plant farm at the National Research Centre, Egypt (latitude 30° 30′ 1.4′′ N, longitude 30° 19′ 10.9′′ E) in May 2022. The plant materials were identified by Prof. Shoukry M. Selim of floriculture, Faculty of Agriculture, Fayoum University. The harvested plant components were washed with tap and distilled water and then dried in a shaded area at room temperature (26 ± 1 °C) for three days. To obtain the herbal oils, 1000 g of leaves from each plant were mixed with 2000 mL of distilled water and subjected to hydro-distillation using a Clevenger apparatus. The resulting oils were dehydrated with anhydrous sodium sulfate and stored in dark bottles at 4 °C until chemical analysis and insecticide formulations.

### Essential oil analysis

*R. officinalis* and *P. anisum* essential oils were analyzed using gas chromatography-mass spectroscopy (GC-MS). The plant essential oils were mixed with diethyl ether. Then 1 µL of the mixture was injected into the GC-MS, Trace GC Ultra coupled with single quadrupole mass spectrometry, and the TG-5MS fused silica capillary column (30 m, 0.25 mm, and 0.1 mm film thickness). Helium gas was used as a carrier gas at a regular flow rate of 1 mL min^− 1^. The injector and MS transfer line were both set at 280 °C while the oven temperature was programmed to start at 50 °C (held for 2 min), increase at a rate of 7 °C per minute to 150 °C, and then further increase at a rate of 5 °C per minute to 270 °C (held for 2 min), before finally increasing at a rate of 3.5 °C per minute to 310 °C (held for 10 min). All components were quantified using a percent comparative peak area. The compounds were tentatively identified based on comparing the retention index of the GC peaks obtained using a homologous series of n-alkanes (C8 to C20) with those reported in the literature^27^. The mass spectra of these components were also matched with those of the GC/MS system’s NIST 08 and WILLY 7 library data.

### Insect rearing

Adult cowpea bruchids (*C. maculatus*) were gathered from a laboratory culture that has been kept free of insecticides since April 2017. A total of 20 female and 20 male bruchids were selected from the culture and placed into 1000 mL glass jars containing 500 g of clean, sterilized cowpea grains (*V. unguiculata* var. Dokki 331) with a moisture content of 9.7% at a temperature of 27 ± 1 °C, relative humidity of 65 ± 5%, and a light-dark photoperiod of 12 h each. The cowpea seeds were frozen for seven days at -13 °C to eliminate any live insects present before use. The jars were covered with cloth and perforated metal lids to prevent the beetles from escaping while allowing sufficient aeration. After seven days, the parental insects were removed by sieving, and the infested cowpea grains were kept in the laboratory until the F1 progeny emerged. For all bioassays, sexed insects between the ages of 1 to 2 days were utilized.

### Preparation and functionalization of zeolites with ZnO nanoparticles

The synthetic zeolites utilized in this study were treated using the procedure described by Youssef, et al.^28^ under microwave conditions of 3.0 M NaOH, 80 °C, and 110 °C for 2 h to yield zeolite powders of zeolite-A and Faujasite-NaX (zeolite-X), respectively. The zeolite products that were previously obtained underwent functionalization through cation exchange processing, which involved doping the zeolite powder in a solution containing zinc. In a typical procedure, one gram of each zeolite type (zeolite-A and zeolite-X) was placed in 100 mL of a 0.1 M zinc chloride solution and stirred gently at 400 rpm for two hours at room temperature. The resulting mixture was then separated by solid/liquid separation using centrifugation at 3600 rpm and repeatedly washed with distilled water until the pH reached 7. The resulting clean powder was dried at 50 °C in an electric oven for two hours and stored in a dry container until characterization could be performed.

### X-ray diffraction of prepared zeolites

The mineral composition of the synthetic product was characterized by the XRD method using BRUKUR D8 ADVANCE with secondary monochromatic beam Cu Kα radiation at KV = 40 and mA = 40.

### Scanning electron microscope and energy dispersive X-ray identification for zeolites

The mineral composition and internal texture of zeolite-A and zeolite-X, along with their ZnO-exchanged materials, were identified by XRD and SEM model Quanta 250 FEG (Field Emission Gun) attached with EDX Unit (Energy Dispersive X-ray analyses), with accelerating voltage 30 KV, magnification 14 up to 1,000,000, and resolution for Gun.1n. Field Electron and Ion Company, Netherlands.

### Toxicity bioassay

#### Efficiency of zeolites

The toxicity of inert dust, including zeolite-A, zeolite-X, and their ZnO-loaded counterparts, against the adult of *C. maculatus* was determined using a direct contact assay. Glass jars with a volume of 500 mL were filled with 100 g of clean cowpeas and treated with zeolites at rates of 250, 500, 750, and 1000 mg/kg. An untreated lot was used as a control. The jars were shaken by hand and then by a rotary mixer for 10 min. Thirty sexed adults of *C. maculatus* (15 males and 15 females) were added to each jar vessel. The jars were tightly closed with cotton cloth to prevent insect escape and ensure proper aeration. Each treatment was replicated four times. Mortality rates were recorded after 2, 5, and 7 days of contact with treated cowpeas. To assess the treatments’ efficacy on progeny production, live and dead individuals were discarded after 7 days, and the jars were kept under the same rearing conditions for 30 days. The number of emerged adults of *C. maculatus* was then counted and expressed as progeny production.

#### Efficiency of zeolites formulated with essential oils

Zeolite-A, zeolite-X, and their ZnO-loaded forms were combined with *R. officinalis* and *P. anisum* essential oils. The formulations were obtained by mixing 100 and 200 mg of each essential oil separately with four types of zeolites (zeolite-A, zeolite-X, Zn-zeolite-A, and Zn-zeolite-X) at 750 and 1000 mg/kg cowpea grains. To ensure even distribution of the zeolite formulations, 1 mL of acetone was added. A control treatment, consisting of cowpea grains treated with 1 mL of acetone only, was included for comparison. After solvent evaporation, the tested insect was exposed to each treatment, and cumulative mortality was recorded after 2, 5, and 7 days. Moreover, the effects of the formulations on insect progeny production were investigated.

#### Joint action studies

The co-toxicity factor (CTF) was used as a criterion to evaluate the combined lethal effect of the essential oils and zeolites. Mortality percentages corresponding to LC_25_ were determined from regression lines. The summation of mortality percentages of zeolites and the essential oils was considered as expected mortality. The tested insect was exposed to pairs of the essential oils and zeolite mixtures at their respective LC_25_ levels. Mortality (%) caused by these mixtures was recorded after 5 days of treatment and considered as observed mortality. A co-toxicity factor was taken as a criterion for evaluation of the joint toxic effect as follows:

CTF = (OM - EM)/EM × 100, where OM is the observed mortality (%) of the mixture and EM (expected mortality) is the sum of mortality (%) caused by each fraction of the mixture when tested individually. A positive CTF value + 20 or higher indicates a synergy effect, a negative CTF value of − 20 or lower indicates an antagonism effect, and values between − 20 and + 20 imply an additive effect^29^.

### Scanning electron microscope for *C. maculatus*

SEM was used to examine *C. maculatus* adults treated with either zeolites or zeolites loaded with zinc at 1000 mg/kg. The dead adults were air-dried for 5 days without any further preparations to ensure the zeolites remained on them. The gold coating process was performed on the samples using Quorum Q 150 ES, United Kingdom, with a 20 nm thick layer of gold applied for 60 s. The untreated and treated dead adults were examined using the TESCAN VEGA 3 electron scan microscope, Czech Republic. The beetle’s whole body’s dorsal and ventral surfaces were observed and captured in photographs.

### Data analysis

Before analysis, the mortality data underwent Arcsin transformation. The data was then separately examined using one-way analysis of variance (ANOVA). Duncan’s New Multiple Range Test was utilized at a significance level of *p* < 0.05 to determine the significance of mean differences. To obtain the LC_50_ values, the concentration–mortality data from laboratory tests was analyzed after 5 d using Probit. All serial concentrations of the essential oils and zeolite combinations had 100 mg/kg of the essential oil. The values of LC_50_ were found to be significantly different if the 95% confidence limit was not crossed. Statistical analysis was performed using XLSTAT Addinsoft 2021.2.2 software.

## Results

### The chemical constituents of essential oils

Table 1 shows the chemical composition of essential oils extracted from *R. officinalis* and *P. anisum* using GC-MS. Based on a fresh plant of *R. officinalis* weight of extract part, the hydro-distillation yielded about 0.3% w/w. Twenty compounds have been identified, representing 97.23% of the essential oil. These compounds were divided into 43.11% monoterpene hydrocarbons (α-thujene, α-pinene, Camphene, β-pinene, myrcene, γ-terpinene, terpinolene, α-phellandrene, and cymene); 47.81% oxygenated monoterpenes (1,8-cineole, linalool, trans-pinocarveol, Camphor, borneol, terpinen-4-ol, and α-terpineol); 4.59% of sesquiterpene hydrocarbons (caryophyllene, aromadendrene, and humulene); 1.72% of ketone (3-octanone). The major compounds of *R. officinalis* essential oil were 1,8-cineole (25.36%), α-pinene (23.75%), Camphor (12.66%), and Camphene (8.19%). For *P. anisum* essential oil, with a yield of 0.35% w/w based on the sample’s fresh weight, twenty-one compounds were recorded, accounting for 96.45%. The essential oil analysis revealed that the oil had a lower quantity of monoterpene hydrocarbons of 3.95% (pinene, carene, limonene, ocimene, and terpinene). The essential oil had rich amounts of oxygenated monoterpenes of 74.71% (linalool, Methyl chavicol, Z-anethole, and E-anethole). The amount of sesquiterpene hydrocarbons was 15.62% (isoledene, longifolene, cedrene, thujopsene, gurjunene, elemene, guaiene, himachalane, and E-isoeugenol), while the oxygenated sesquiterpenes recorded 2.17% (cis-sesquisabinene hydrate, spathulenol, and geranyl isovalerate). *P. anisum* essential oil contained a high percentage of E-anethole (64.23%), followed by methyl chavicol (8.69%) and longifolene (5.08%). The two oils also differed in their terpene hydrocarbon content. *R. officinalis* had a higher proportion of monoterpene hydrocarbons (43.11%) than *P. anisum* (3.95%). In contrast, *P. anisum* has a higher percentage of oxygenated monoterpenes (74.71%) than *R. officinalis* (47.81%).

**Table 1:** Chemical composition of *Rosmarinus officinalis* and *Pimpinella anisum* essential oils.

*R*. officinalis	RT^a^	RI_exp_	RI_lit_	Percentage	*P*. anisum	RT^a^	RI_exp_	RI_lit_	Percentage
α-Thujene	5.32	925	924	0.28	α-Pinene	5.55	934	932	0.38
α-Pinene	5.55	934	932	23.75	δ-3-Carene	7.61	1009	1008	0.84
Camphene	5.96	949	946	8.19	Limonene	8.19	1025	1024	1.25
β-Pinene	6.64	975	974	5.21	E-β-Ocimene	8.92	1046	1044	0.75
3-Octanone	6.89	985	979	1.72	γ-Terpinene	9.28	1056	1054	0.73
Myrcene	7.03	990	988	1.42	Linalool	10.72	1098	1095	0.67
α-Phellandrene	7.35	1002	1002	0.34	Methyl Chavicol	14.77	1197	1195	8.69
Cymene	8.22	1026	1022	2.53	Z-Anethole	16.96	1249	1249	1.12
1,8-Cineole	8.36	1030	1026	25.36	E-Anethole	18.43	1284	1282	64.23
γ-Terpinene	9.28	1056	1054	1.03	Isoledene	22.28	1376	1374	1.38
Terpinolene	10.38	1088	1086	0.36	Longifolene	23.52	1405	1407	5.08
Linalool	10.72	1098	1095	0.62	α-Cedrene	23.76	1412	1410	0.64
Trans-Pinocarveol	12.29	1136	1135	0.21	Cis-Thujopsene	24.46	1429	1429	1.45
Camphor	12.58	1144	1141	12.66	β-Gurjunene	24.65	1434	1431	0.86
Borneol	13.59	1168	1165	4.39	γ-Elemene	24.69	1435	1434	0.73
Terpinen-4-ol	13.96	1177	1174	0.85	α-Guaiene	24.80	1437	1437	0.52
α-Terpineol	14.31	1186	1186	3.72	E-Isoeugenol	25.30	1450	1448	3.58
Caryophyllene	24.06	1419	1417	2.47	α-Himachalene	25.42	1453	1449	1.38
Aromadendrene	24.87	1439	1439	1.65	Cis-Sesquisabinene Hydrate	29.06	1545	1542	0.51
α-Humulene	25.46	1454	1452	0.47	Spathulenol	30.36	1578	1577	0.84
Monoterpene hydrocarbons				43.11	Geranyl isovalerate	31.45	1607	1606	0.82
Oxygenated monoterpenes				47.81	Monoterpene hydrocarbons				3.95
Sesquiterpene hydrocarbons				4.59	Oxygenated monoterpenes				74.71
Oxygenated sesquiterpenes				0.00	Sesquiterpene hydrocarbons				15.62
Other				1.72	Oxygenated sesquiterpenes				2.17
Total				97.23	Total				96.45

^a^RI: Retention indices determined using the homologous series of n-alkanes (C8-C22).

### Characterizations of the synthetic zeolites

**Fig. 1:**
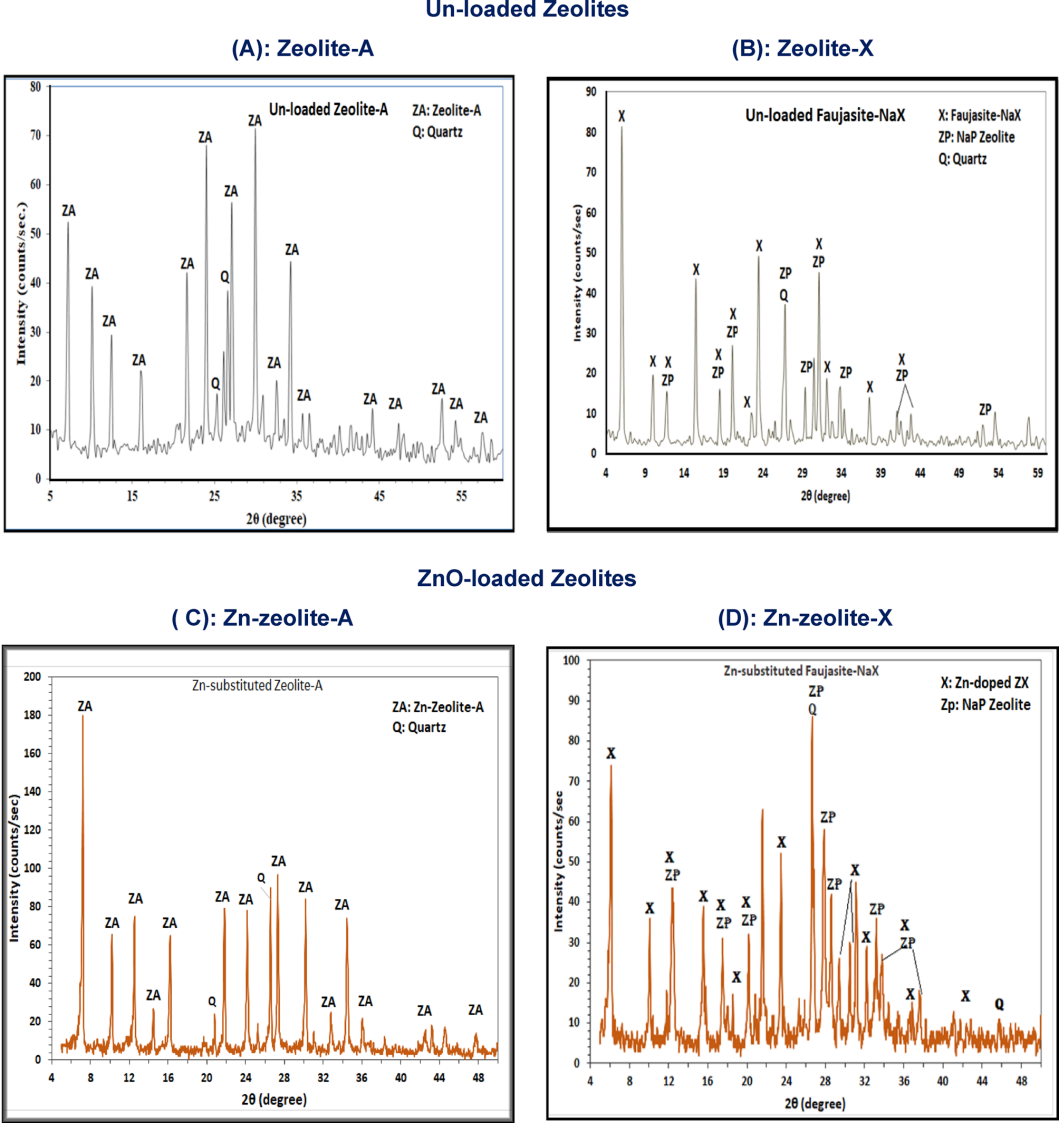
XRD for the synthetic zeolites before and after ZnO functionalization.

Figure 1A and B presents the parent zeolite (zeolite-A and zeolite-X). Zeolites mineral profiles were compared to the XRD database and showed perfect matching with PDF card # 73-2340, with Na_12_Al_12_Si_12_O_48_.27H_2_O for zeolite-A (Fig. 1A), and PDF # 39-1380 (1), with Na_2_Al_2_Si_4_O_12_.8H_2_O composition for zeolite-X (Fig. 1B), in respective order. The distinct, sharp, and complete set of both zeolites’ peaks implied good crystallinity. Notably, the synthetic product contains some residue of quartz mineral, which was traced back to the kaolin precursor from which they were formed. Meanwhile, Faujasite-NaX showed a small number of nanoparticle zeolite peaks that seemed to accompany the originally developed zeolite-X material, and this could be seen in light of the similarity in preparation conditions of both zeolites.

Figure 1C and D shows XRD details for Zn-doped zeolites. Both charts compared to the standard references of the PDF-2 database and confirmed the evolution of Zn-doped types of Zn-zeolite-A and Zn-zeolite-X, having respective chemical compositions of Na_50_Zn_23_Al_96_Si_96_O_384_.216H_2_O (Fig. 1C) and (Zn, Na)_2_Al_2_Si_2.5_O_9_.72H_2_O (Fig. 1D), respectively. As shown in Fig. 1 (C-D) the XRD patterns for Zn-containing phases indicated sharper peaks with higher intensities than those recorded for their un-doped forms (Fig. 1A and B). In addition, zeolite-X implies the presence of minor amounts of zeolite nanoparticles as a secondary accompanying phase that can develop in the zeolite mixtures. This indicates very high crystallinity of many pure phases with no residues of the amorphous metakaolin precursor, which was preserved within the synthetic zeolite powders and appeared in the XRD patterns in the form of a humpy background in Fig. 1 (A and B).

### Internal structure testing (SEM and EDS)

**Fig. 2:**
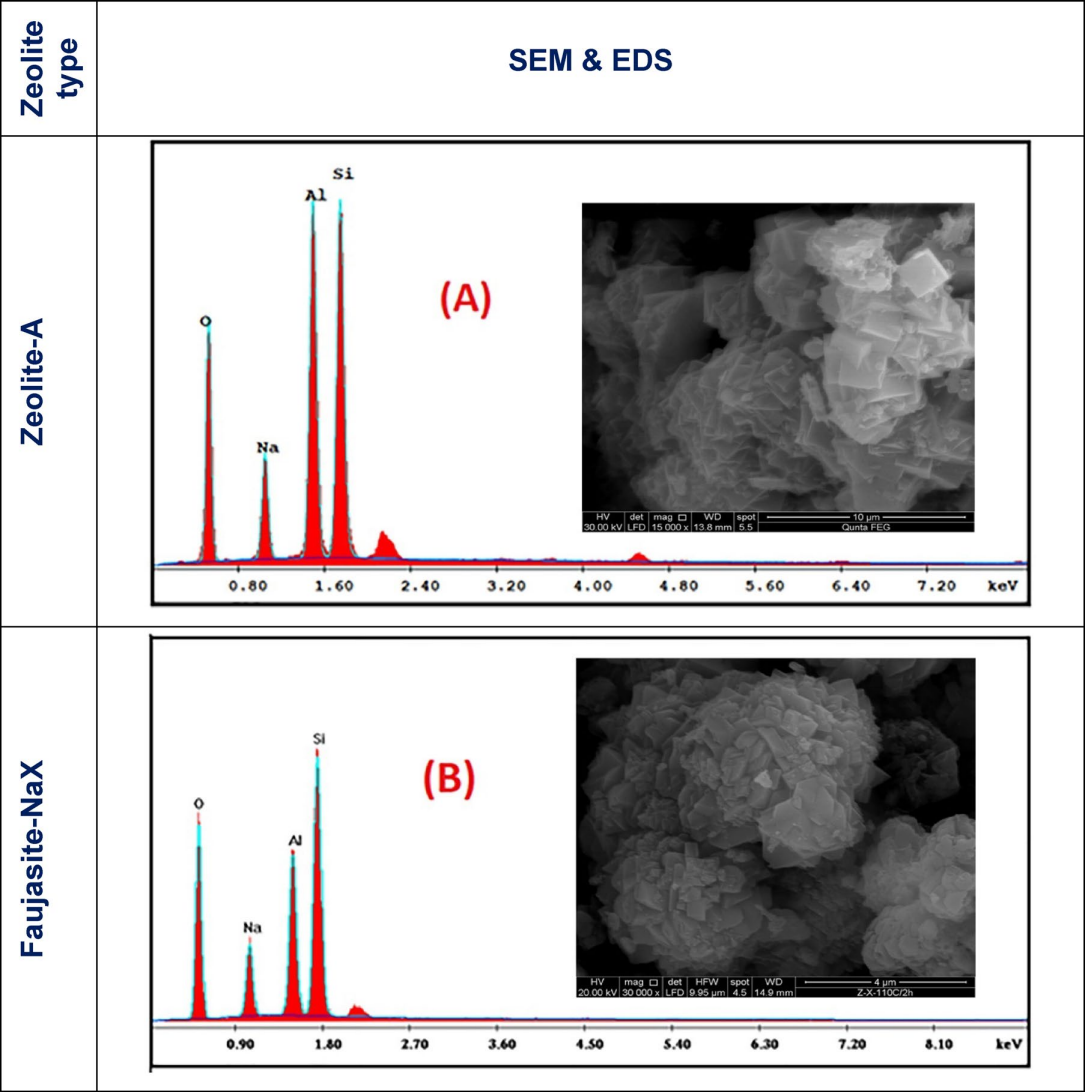
SEM and EDS for the as-synthesized zeolites. (**A**). zeolite-A, and (**B**) zeolite-X.

The internal textural analysis of zeolite product and its chemical microanalysis give a clear idea about the characteristic morphology and elemental contents of the contained crystals. The internal crystalline texture and the elemental micro-chemical analysis of the dry, unfunctionalized zeolite-A and zeolite-X products were examined using the SEM and EDS tools. The obtained data are given in Fig. 2A and B and Table 2. As can be noticed from Fig. 2A, zeolite-A exhibited its distinctive cubic-shaped crystal form with uniform grain particles in the range of 1–3 μm in size.

**Table 2:** Energy dispersive X-ray spectroscopy micro-chemical analysis for Zn-doped products.

Element	Zeolite-A	Atomic %	Zeolite-X	Atomic %
	Weight%		Weight%	
O K	53.78	70.24	54.13	69.44
Na K	0.01	0.03	0.01	0.01
Al K	16.46	12.75	14.13	10.75
Si K	17.65	13.13	23.62	17.25
Zn K	12.1	3.87	8.11	2.55
Total	100	100	100	100

**Fig. 3:**
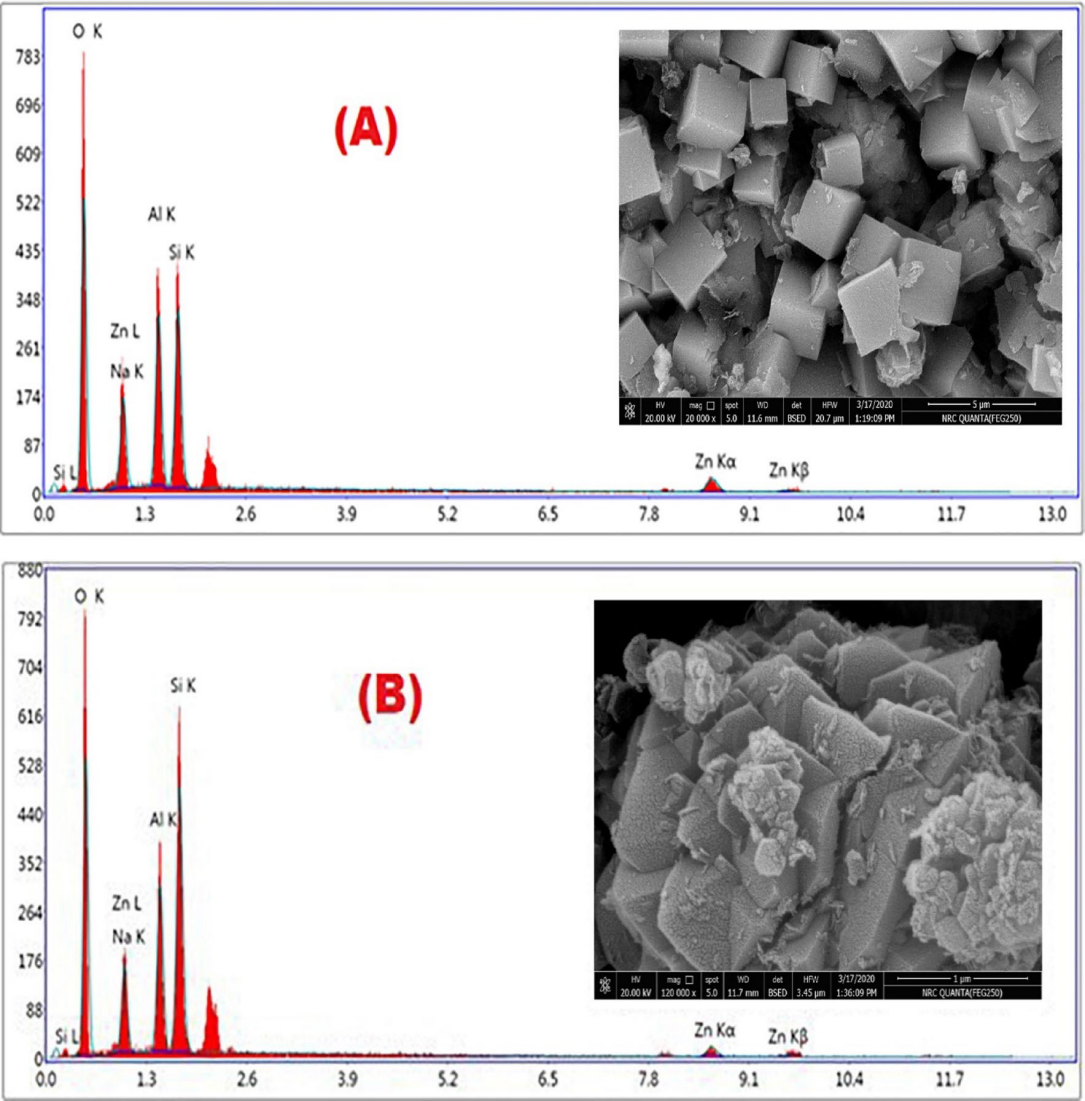
SEM and EDS microanalysis for the synthetic zeolites after Zn-functionalization. (**A**) Zn-zeolite-A, (**B**) Zn-zeolite-X.

Figure 2B presents the obtained product of Faujasite-NaX (zeolite-X). The micrograph monitors large crystals with pyramidal epics of less than 1 μm in size, accompanied by an ample amount of zeolite nanoparticles (10–15%) and some scattered quartz particles (< 5%). The former SEM result for both zeolites is consistent with the previous XRD data. Table 2 demonstrates the elemental composition of the un-doped zeolites, where the calculated average Si/Al ratios of the crystal composition were 1.11 and 1.75 for zeolite-A and zeolite-X, respectively. Figure 3A and B monitors the SEM morphologies of the obtained zeolite-produced powders after the exchange of their sodium constituents by zinc. The micro-chemical composition is given in Table 2. Obviously, there were no destructive changes in the crystal configuration for both zeolites after doping since the crystal identities were preserved without shape alteration. The only notice was the clear crystal faces and edges. The results of the EDS analysis were collected from an average of three measurement detections for the crystal surfaces of three different crystal generations of the same zeolite species. The respective atomic ratio of Si/Al for zeolite-A was 1.07, and for zeolite-X was 1.6 (Table 2).

### Efficiency of zeolite nanoparticles on *C. maculatus*

The mortality of *C. maculatus* treated with zeolites at different durations is presented in Table 3. The mortality of the beetles increased with increasing concentration and duration of exposure for all treatments. The results also showed significant differences in mortality between the different types and concentrations of zeolites at each time interval.

The highest mortality rate achieved by zeolite-X was 48.3% after 7 days at 1000 mg/kg, while it was 43.3% for zeolite-A at the same concentration and time interval. The highest mortality was observed for Zn-zeolite-A (51.7%) at 1000 mg/kg after 7 days. Zeolite-X outperformed zeolite-A in insecticidal activity against *C. maculatus*, while zeolite-A loaded with zinc surpassed Zn-zeolite-X in insecticidal efficacy.

**Table 3:** Mortality of *Callosobruchus maculatus* exposed for 2, 5, and 7 days to Cowpea treated with zeolites (zeolite-X, zeolite-A, and zinc-loaded zeolite) at different concentrations.

Treatments (concentration, mg/kg)	Mean mortality (%) ± SE		
	2 days	5 days	7 days
Cont.	3.3 ± 3.3 gh	8.3 ± 1.7 i	8.3 ± 1.7 i
Zeolite-X (250)	6.7 ± 3.3 fgh	16.7 ± 3.3 gh	28.3 ± 1.7 gh
Zeolite-X (500)	15.0 ± 2.9 cdef	26.7 ± 1.7 def	33.3 ± 1.7 defgh
Zeolite-X (750)	18.3 ± 1.7 abcde	30.0 ± 0.0 bcde	38.3 ± 1.7 cde
Zeolite-X (1000)	25.0 ± 2.9 ab	38.3 ± 1.7 a	48.3 ± 1.7 ab
Zeolite-A (250)	1.7 ± 1.7 h	11.7 ± 1.7 hi	26.7 ± 3.3 h
Zeolite-A (500)	11.7 ± 1.7 efg	23.3 ± 1.7 ef	30.0 ± 2.9 fgh
Zeolite-A (750)	13.3 ± 1.7 def	26.7 ± 1.7 def	36.7 ± 1.7 def
Zeolite-A (1000)	18.3 ± 1.7 abcde	35.0 ± 2.9 abc	43.3 ± 1.7 bc
Zn-zeolite-X (250)	6.7 ± 3.3 fgh	15.0 ± 2.9 gh	28.3 ± 1.7 gh
Zn-zeolite-X (500)	16.7 ± 4.4 bcde	28.3 ± 1.7 cde	33.3 ± 1.7 defgh
Zn-zeolite-X (750)	21.7 ± 1.7 abcd	30.0 ± 0.0 bcde	35.0 ± 2.9 defg
Zn-zeolite-X (1000)	26.7 ± 1.7 a	36.7 ± 1.7 ab	46.7 ± 1.7 ab
Zn-zeolite-A (250)	6.7 ± 3.3 fgh	20.0 ± 5.0 fg	31.7 ± 3.3 efgh
Zn-zeolite-A (500)	16.7 ± 4.4 bcde	30.0 ± 0.0 bcde	36.7 ± 1.7 def
Zn-zeolite-A (750)	23.3 ± 1.7 abc	33.3 ± 1.7 abcd	40.0 ± 0.0 cd
Zn-zeolite-A (1000)	26.7 ± 1.7 a	40.0 ± 0.0 a	51.7 ± 1.7
Df	16, 34	16, 34	16, 34
F value	8.91	19.34	23.74
*P*	< 0.0001	< 0.0001	< 0.0001

The mean within a column that shares the same letter is not significantly different at *p* < 0.05.

### Efficiency of zeolite nanoparticles and *R. officinalis* combinations on *C. maculatus*

The mortality of zeolite and *R. officinalis* combinations after 2, 5, and 7 days against the tested insect is elucidated in Table 4. The concentration- and time-dependent mortality was evident in all treatments compared to the control group that had minimal mortality (3.3%) even after seven days of exposure. Use of *R. officinalis* essential oil at a single dose produced moderate insecticidal activity with the higher dose (200 mg/kg) causing a higher mortality of 63.3% on the seventh day, compared to 43.3% at the lower concentration (100 mg/kg). When *R. officinalis* essential oil was used together with zeolites, a high improvement in insecticidal activity was observed. In most cases, the higher the concentration of the essential oil and the zeolites, the higher the mortality. The mixtures of the high concentration of *R. officinalis* (200 mg/kg) with any of the tested zeolites at 750 or 1000 mg/kg were the most effective. Interestingly, several treatments (*R. officinalis* (200 mg/kg) combined with Zeolite-X (both 750 and 1000 mg/kg), Zeolite-A (1000 mg/kg), Zn-zeolite-X (1000 mg/kg), and Zn-zeolite-A (both 750 and 1000 mg/kg)) reached 100% mortality by day seven. The fastest and most efficient treatment was the combination of *R. officinalis* (200 mg/kg) and Zn-zeolite-A (1000 mg/kg) that led to 100% mortality in only five days of exposure.

**Table 4:** Mortality of *Callosobruchus maculatus* exposed for 2, 5, and 7 days to Cowpea treated with *R. officinalis* essential oil applied alone and in combinations with zeolites (zeolite-X, zeolite-A, and zinc-loaded zeolite) at different concentrations.

Treatments (concentration, mg/kg)	Mean mortality (%) ± SE		
	2 days	5 days	7 days
Cont.	0.0 ± 0.0 k	0.0 ± 0.0 m	3.3 ± 1.7 h
RO (100)	21.7 ± 4.4 j	33.3 ± 3.3 l	43.3 ± 3.3 g
RO (200)	43.3 ± 3.3 hi	50.0 ± 0.0 k	63.3 ± 3.3 f
RO (100) + Zeolite-X (750)	48.3 ± 1.7 gh	60.0 ± 2.9 ij	73.3 ± 4.4 de
RO (100) + Zeolite-X (1000)	55.0 ± 2.9 efg	73.3 ± 1.7 fgh	83.3 ± 1.7 bc
RO (200) + Zeolite-X (750)	68.3 ± 1.7 cd	88.3 ± 1.7 cd	100.0 ± 0.0 a
RO (200) + Zeolite-X (1000)	73.3 ± 3.3 bc	95.0 ± 5.0 abc	100.0 ± 0.0 a
RO (100) + Zeolite-A (750)	38.3 ± 3.3 i	55.0 ± 2.9 jk	70.0 ± 5.0 ef
RO (100) + Zeolite-A (1000)	51.7 ± 3.3 fg	73.3 ± 4.4 fgh	80.0 ± 2.9 bcd
RO (200) + Zeolite-A (750)	61.7 ± 1.7 de	80.0 ± 0.0 ef	93.3 ± 1.7 a
RO (200) + Zeolite-A (1000)	68.3 ± 1.7 cd	88.3 ± 1.7 cd	100.0 ± 0.0 a
RO (100) + Zn-zeolite-X (750)	38.3 ± 1.7 i	56.7 ± 1.7 jk	76.7 ± 3.3 cde
RO (100) + Zn-zeolite-X (1000)	56.7 ± 3.3 ef	70.0 ± 2.9 gh	85.0 ± 2.9 b
RO (200) + Zn-zeolite-X (750)	66.7 ± 3.3 cd	83.3 ± 1.7 de	95.0 ± 2.9 a
RO (200) + Zn-zeolite-X (1000)	80.0 ± 0.0 ab	98.3 ± 1.7 ab	100.0 ± 0.0 a
RO (100) + Zn-zeolite-A (750)	41.7 ± 3.3 hi	66.7 ± 3.3 hi	78.3 ± 4.4 bcd
RO (100) + Zn-zeolite-A (1000)	58.3 ± 4.4 ef	75.0 ± 0.0 fg	83.3 ± 1.7 bc
RO (200) + Zn-zeolite-A (750)	71.7 ± 1.7 c	91.7 ± 1.7 bc	100.0 ± 0.0 a
RO (200) + Zn-zeolite-A (1000)	81.7 ± 1.7 a	100.0 ± 0.0 a	100.0 ± 0.0 a
Df	18, 38	18, 38	18, 38
F value	62.12	104.01	88.70
*P*	< 0.0001	< 0.0001	< 0.0001

The mean within a column that shares the same letter is not significantly different at *p* < 0.05.

RO, *Rosmarinus officinalis* essential oil.

### Efficiency of zeolite nanoparticles and *P. anisum* combinations on *C. maculatus*

The mortality of zeolite and *P. anisum* combinations after 2, 5, and 7 days against the tested insect is presented in Table 5. The findings indicate that *P. anisum* and *R. officinalis* essential oils alone or in combination with zeolites were found to significantly increase the mortality of *C. maculatus* compared to the control. *P. anisum* was more effective than *R. officinalis* at the same concentrations. For example, *P. anisum* (200 mg/kg) produced 63.3% mortality after 2 days, compared to 43.3% in *R. officinalis* (200 mg/kg). This was consistent throughout the exposure periods, and the *P. anisum* treatments tended to produce more rapid and more severe lethal effects. The synergistic mixtures of *P. anisum* or *R. officinalis* with zeolites also increased the mortality, especially at increased concentrations (1000 mg/kg). It is worth noting that *P. anisum*-based formulations, such as *P. anisum* (200) + Zn-zeolite-A (1000) was able to kill 100% of the larvae in 5 days whereas the most effective *R. officinalis* combination, *R. officinalis* (200) + Zn-zeolite-A (1000) took 7 days to kill the larvae to the same extent.

**Table 5:** Mortality of *Callosobruchus maculatus* exposed for 2, 5, and 7 days to Cowpea treated with *Pimpinella anisum* essential oil applied alone and in combinations with zeolites (zeolite-X, zeolite-A, and zinc loaded zeolite) at different concentrations.

Treatments (concentration, mg/kg)	Mean mortality (%) ± SE		
	2 days	5 days	7 days
Cont.	1.7 ± 1.7 h	3.3 ± 1.7 h	5.0 ± 0.0 g
PA (100)	43.3 ± 3.3 g	53.3 ± 3.3 g	66.7 ± 3.3 f
PA (200)	63.3 ± 3.3 de	73.3 ± 3.3 def	80 ± 5.8 de
PA (100) + Zeolite-X (750)	58.3 ± 1.7 ef	71.7 ± 1.7 ef	81.7 ± 1.7 cde
PA (100) + Zeolite-X (1000)	68.3 ± 1.7 d	78.3 ± 1.7 cde	86.7 ± 1.7 bcd
PA (200) + Zeolite-X (750)	83.3 ± 1.7 bc	93.3 ± 1.7 ab	100.0 ± 0.0 a
PA (200) + Zeolite-X (1000)	88.3 ± 1.7 ab	100.0 ± 0.0 a	100.0 ± 0.0 a
PA (100) + Zeolite-A (750)	60.0 ± 0.0 ef	68.3 ± 1.7 f	75.0 ± 2.9 e
PA (100) + Zeolite-A (1000)	68.3 ± 1.7 d	80.0 ± 5.0 cd	83.3 ± 4.4 cd
PA (200) + Zeolite-A (750)	81.7 ± 1.7 bc	90.0 ± 2.9 b	98.3 ± 1.7 a
PA (200) + Zeolite-A (1000)	83.3 ± 1.7 bc	93.3 ± 1.7 ab	100.0 ± 0.0 a
PA (100) + Zn-zeolite-X (750)	56.7 ± 1.7 ef	68.3 ± 1.7 f	83.3 ± 1.7 cd
PA (100) + Zn-zeolite-X (1000)	68.3 ± 4.4 d	81.7 ± 1.7 c	91.7 ± 1.7 b
PA (200) + Zn-zeolite-X (750)	80.0 ± 2.9 c	95.0 ± 2.9 ab	100.0 ± 0.0 a
PA (200) + Zn-zeolite-X (1000)	86.7 ± 1.7 abc	96.7 ± 1.7 ab	100.0 ± 0.0 a
PA (100) + Zn-zeolite-A (750)	53.3 ± 1.7 f	68.3 ± 3.3 f	81.7 ± 1.7 cde
PA (100) + Zn-zeolite-A (1000)	63.3 ± 1.7 de	76.7 ± 1.7 cde	88.3 ± 1.7 bc
PA (200) + Zn-zeolite-A (750)	85.0 ± 0.0 abc	96.7 ± 1.7 ab	100.0 ± 0.0 a
PA (200) + Zn-zeolite-A (1000)	91.7 ± 4.4 a	100.0 ± 0.0 a	100.0 ± 0.0 a
Df	18, 38	18, 38	18, 38
F value	81.78	91.11	100.15
*P*	< 0.0001	< 0.0001	< 0.0001

The mean within a column that shares the same letter is not significantly different at *p* < 0.05.

PA, *Pimpinella anisum* essential oil.

### Effect of zeolite nanoparticles on progeny production

The mortality of progeny of *C. maculatus* exposed to zeolites with and without loaded zinc is recorded in Table 6. The results showed that all zeolite treatments caused a significant increase in the mortality of *C. maculatus* offspring compared to the control. As the concentration of zeolite increases, the mean number of progeny decreases. None of the concentrations applied could suppress the progeny production. All zeolite treatments showed moderate effects on the mortality of progeny of *C. maculatus.* Zeolite-X and zeolite-A loaded zinc were the most effective treatments with mortality of offspring of 48.43 and 49.64%, respectively at the highest application rate of 1000 mg/kg.

**Table 6:** Mortality of progeny of *Callosobruchus maculatus* treated with zeolites (zeolite-X, zeolite-A, and zinc-loaded zeolite) at different concentrations.

Treatments (concentration, mg/kg)	Mean no. of progeny ± SE	Mean no. of dead insects ± SE	% Mortality of progeny ± SE
Cont.	127.0 ± 5.6 a	4.3 ± 0.3	3.38 ± 0.1 e
Zeolite-X (750)	71.3 ± 3.5 c	27.3 ± 0.9	38.28 ± 1.7 cd
Zeolite-X (1000)	54.3 ± 2.3 ef	26.3 ± 1.8	48.43 ± 1.2 ab
Zeolite-A (750)	82.7 ± 2.0 b	30.0 ± 1.7	36.27 ± 1.8 cd
Zeolite-A (1000)	62.7 ± 2.9 cde	26.7 ± 2.0	42.58 ± 1.4 bc
Zn-zeolite-X (750)	59.7 ± 2.7 de	23.0 ± 1.5	38.52 ± 1.2 cd
Zn-zeolite-X (1000)	41.7 ± 0.3 g	20.7 ± 0.9	45.51 ± 4.4 ab
Zn-zeolite-A (750)	65.0 ± 2.9 cd	21.3 ± 1.5	32.76 ± 1.8 d
Zn-zeolite-A (1000)	49.0 ± 1.5 fg	22.3 ± 1.5	49.64 ± 2.4 a
Df	8, 18		8, 18
F value	71.68		44.66
*P*	< 0.0001		< 0.0001

The mean within a column that shares the same letter is not significantly different at *p* < 0.05.

### Effect of zeolite nanoparticles and *R. officinalis* combinations on progeny production

Data presented in Table 7 show the mortality of progeny of *C. maculatus* treated with zeolite and *R. officinalis* combinations. All treatments significantly reduced the mean number of progeny and increased the percentage of offspring mortality of *C. maculatus* compared to the control. The treatment with *R. officinalis* essential oil (RO) alone at 100 mg/kg resulted in a mean number of progeny of 42, which is significantly lower than the control group (129). Additionally, the mortality of the progeny under this treatment was 35.7%. Increasing the concentration of *R. officinalis* essential oil to 200 mg/kg further reduced the mean number of progeny to 21 and increased the mortality percentage to 55.2%. The combination of zeolite and *R. officinalis* essential oil increased the mortality of progeny compared with zeolite alone. Zeolites loaded with zinc and *R. officinalis* oil mixtures could suppress progeny production at 200 mg/kg of the essential oil and 1000 mg/kg of zeolite.

**Table 7:** Mortality of progeny of *Callosobruchus maculatus* treated with *R. officinalis* essential oil applied alone and in combinations with zeolites (zeolite-X, zeolite-A, and zinc-loaded zeolite) at different concentrations.

Treatments (Concentration, mg/kg)	Mean no. of progeny ± SE	Mean no. of dead insects ± SE	% Mortality of progeny ± SE
Cont.	129.0 ± 3.2 a	4.0 ± 1.0	3.1 ± 0.7 p
RO (100)	42.0 ± 1.2 b	15.0 ± 1.2	35.7 ± 3.7 o
RO (200)	21.0 ± 1.5 fghi	11.6 ± 0.9	55.2 ± 2.2 klm
RO (100) + Zeolite-X (750)	22.3 ± 1.2 fg	16.7 ± 0.9	75.5 ± 8.0 defg
RO (100) + Zeolite-X (1000)	18.3 ± 0.9 ghijk	15.0 ± 1.0	81.7 ± 2.1 cdef
RO (200) + Zeolite-X (750)	7.0 ± 0.6 nop	6.0 ± 0.6	85.5 ± 1.2 bcde
RO (200) + Zeolite-X (1000)	4.3 ± 0.7 p	4.3 ± 0.7	100.0 ± 0.0 a
RO (100) + Zeolite-A (750)	24.0 ± 1.5 f	16.3 ± 0.3	68.6 ± 4.7 ghij
RO (100) + Zeolite-A (1000)	17.3 ± 1.3 hijk	13.7 ± 1.7	78.3 ± 3.3 cdefg
RO (200) + Zeolite-A (750)	10.0 ± 0.6 mno	8.7 ± 0.3	86.9 ± 2.6 bcd
RO (200) + Zeolite-A (1000)	5.7 ± 0.9 op	5.0 ± 0.6	89.7 ± 5.2 abc
RO (100) + Zn-zeolite-X (750)	23.7 ± 1.9 f	18.3 ± 1.9	77.5 ± 5.0 defg
RO (100) + Zn-zeolite-X (1000)	15.0 ± 1.5 jkl	12.3 ± 1.3	82.2 ± 1.8 bcdef
RO (200) + Zn-zeolite-X (750)	7.0 ± 0.6 nop	6.0 ± 1.0	84.7 ± 9.7 bcde
RO (200) + Zn-zeolite-X (1000)	0.0 ± 0.0 q	–	–
RO (100) + Zn-zeolite-A (750)	22.0 ± 2.9 fgh	16.3 ± 2.4	74.1 ± 3.0 efgh
RO (100) + Zn-zeolite-A (1000)	14.3 ± 2.4 klm	11.7 ± 2.3	80.5 ± 3.9 cdef
RO (200) + Zn-zeolite-A (750)	4.3 ± 0.3 p	4.3 ± 0.3	100.0 ± 0.0 a
RO (200) + Zn-zeolite-A (1000)	0.0 ± 0.0 q	–	–
Df	34, 70		32, 66
F value	205.19		33.43
*P*	< 0.0001		< 0.0001

The mean within a column that shares the same letter is not significantly different at *p* < 0.05.

### Effect of zeolite nanoparticles and *P. anisum* oil combinations on progeny production

The results of Table 8 demonstrate that the progeny of *C. maculatus* mortality was greatly affected using *P. anisum* essential oil, either alone or in combination with various zeolites. The progeny mortality rate of the control group was low at 2.1%. On the other hand, the mortality rate was significantly higher when *P. anisum* essential oil at 100 and 200 mg/kg was used alone (55.0 and 64.6%, respectively). An interesting synergistic effect was also found when *P. anisum* essential oil was used together with zeolites. The combination of *P. anisum* at 200 mg/kg with different zeolites was the most effective treatments. A complete mortality was observed with *P. anisum* (200 mg/kg) and Zeolite-A (750 mg/kg). Moreover, a number of combinations such as *P. anisum* (200 mg/kg) and either Zeolite-X (750 and 1000 mg/kg), Zeolite-A (1000 mg/kg), or zinc-loaded zeolites (both 750 and 1000 mg/kg) totally inhibited the development of insect progeny.

**Table 8:** Mortality of progeny of *Callosobruchus maculatus* treated with *P. anisum* essential oil applied alone and in combinations with zeolites (zeolite-X, zeolite-A, and zinc-loaded zeolite) at different concentrations.

Treatments (Concentration, mg/kg)	Mean no. of progeny ± SE	Mean no. of dead insects ± SE	% Mortality of progeny ± SE
Cont.	127.0 ± 5.6 a	2.7 ± 0.7	2.1 ± 0.4 h
PA (100)	34.0 ± 2.1 b	18.7 ± 1.5	55.0 ± 1.0 g
PA (200)	11.3 ± 0.9 f	7.3 ± 0.3	64.6 ± 2.5 f
PA (100) + Zeolite-X (750)	17.3 ± 1.5 cde	14.3 ± 1.2	82.7 ± 2.7 de
PA (100) + Zeolite-X (1000)	11.3 ± 0.9 f	10.0 ± 0.6	88.5 ± 2.0 bcd
PA (200) + Zeolite-X (750)	0.0 ± 0.0 g	–	–
PA (200) + Zeolite-X (1000)	0.0 ± 0.0 g	–	–
PA (100) + Zeolite-A (750)	20.3 ± 1.5 c	15.7 ± 0.7	77.4 ± 3.0 e
PA (100) + Zeolite-A (1000)	15.0 ± 1.2 def	13.0 ± 1.0	86.8 ± 3.6 bcd
PA (200) + Zeolite-A (750)	3.3 ± 0.3 g	3.3 ± 0.3	100.0 ± 0.0 a
PA (200) + Zeolite-A (1000)	0.0 ± 0.0 g	–	–
PA (100) + Zn-zeolite-X (750)	19.0 ± 1.2 cd	16.0 ± 1.0	84.3 ± 2.6 cd
PA (100) + Zn-zeolite-X (1000)	13.3 ± 0.7 ef	12.0 ± 0.6	90.1 ± 2.2 bc
PA (200) + Zn-zeolite-X (750)	0.0 ± 0.0 g	–	–
PA (200) + Zn-zeolite-X (1000)	0.0 ± 0.0 g	–	–
PA (100) + Zn-zeolite-A (750)	17.3 ± 1.8 cde	14.7 ± 1.3	84.9 ± 2.6 cd
PA (100) + Zn-zeolite-A (1000)	10.3 ± 1.2 f	9.7 ± 1.5	92.8 ± 3.7 b
PA (200) + Zn-zeolite-A (750)	0.0 ± 0.0 g	–	–
PA (200) + Zn-zeolite-A (1000)	0.0 ± 0.0 g	–	–
Df	18, 38		18, 38
F value	317.63		472.86
P	< 0.0001		< 0.0001

The mean within a column that shares the same letter is not significantly different at *p* < 0.05.

### Toxicity of zeolites, essential oils, and their combinations on *C. maculatus*

The results of contact toxicity of zeolites, essential oils, and their combinations against *C. maculatus* are recorded in Table 9. *P. anisum* oil exhibited higher toxicity than *R. officinalis* oil against the tested insect, with LC_50_ values of 126 and 200 mg/kg, respectively. Zeolite-X and zeolite-A had high LC_50_ values (1407 and 1658 mg/kg, respectively), suggesting lower toxicity than essential oils. Zinc loading in zeolite-X and zeolite-A showed a slight increase in toxicity. The combinations of essential oils (*R. officinalis* and *P. anisum*) with zeolites (zeolite-X, zeolite-A, zeolite-X loaded with zinc, and zeolite-A loaded with zinc) significantly lowered LC_50_ and LC_95_ values compared to the individual components alone. The LC_50_ values for the combinations ranged from 161 to 306 mg/kg. The combination of *P. anisum* oil with zeolite-A loaded with zinc exhibited the lowest LC_50_ value (161 mg/kg), suggesting the highest toxicity among the tested combinations..

**Table 9:** Toxicity of zeolites (zeolite-X, zeolite-A, and zinc-loaded zeolite), essential oils, and their combinations against *Callosobruchus maculatus*.

Treatments	LC_50_ (mg/kg) (95% confidence limits)	LC_95_ (mg/kg) (95% confidence limits)	Slop ± (SE)	Intercept ± (SE)	(χ2)	df
RO oil	200 (175–220)	407 (342–565)	5.32 ± 0.93	− 12.23 ± 2.20	9.32	13
PA oil	126 (90–157)	331 (281–420)	0.01 ± 0.01	− 1.01 ± 0.23	4.38	13
Zeolite-X	1407 (1158–1727)	15,777 (10063–30151)	1.56 ± 0.15	− 4.93 ± 0.48	10.63	19
Zeolite-A	1658 (1366–2046)	18,125 (11499–34807)	1.58 ± 0.15	− 5.09 ± 0.48	7.04	19
Zn-zeolite-X	1305 (1072–1598)	14,874 (9516–28343)	1.55 ± 0.15	− 4.85 ± 0.48	9.38	19
Zn-zeolite-A	1245 (1023–1521)	13,986 (9014–26346)	1.56 ± 0.15	− 4.84 ± 0.48	10.16	19
RO + Zeolite-X	280 (191–355)	1354 (949–2746)	2.40 ± 0.45	− 5.89 ± 1.20	3.82	13
RO + Zeolite-A	306 (210–388)	1629 (1097–3678)	2.26 ± 0.43	− 5.64 ± 1.17	2.74	13
RO + Zn-zeolite-X	259 (169–333)	1286 (901–2641)	2.36 ± 0.45	− 5.71 ± 1.21	3.61	13
RO + Zn-zeolite-A	236 (143–310)	1252 (869–2698)	2.27 ± 0.46	− 5.39 ± 1.22	3.37	13
PA + Zeolite-X	208 (120–271)	875 (620–1976)	2.63 ± 0.61	− 6.11 ± 1.57	4.65	10
PA + Zeolite-A	227 (141–291)	947 (669–2100)	2.65 ± 0.59	− 6.24 ± 1.52	3.22	10
PA + Zn-zeolite-X	190 (96–256)	875 (610–2187)	2.48 ± 0.61	− 5.66 ± 1.57	4.28	10
PA + Zn-zeolite-A	161 (61–229)	809 (559–2267)	2.34 ± 0.63	− 5.18 ± 1.62	4.32	10

χ^2^ = Chi square value; RO = *Rosmarinus officinalis* oil; PA = *Pimpinella anisum* oil.

### Combined toxic effect of essential oils and zeolites

The results in Table 10 show the effectiveness of two essential oils, *R. officinalis* and *P. anisum*, in combination with different zeolite substrates (natural and Zn-loaded Zeolite). All the binary combinations showed positive co-toxicity factors of 20.9 to 30.0, which is a.

sign of synergism. The combination *P. anisum* + Zn-zeolite-A was the one that produced the highest observed mortality (65%), and the highest co-toxicity factor (30), thus indicating a very strong joint effect. As a rule, the Zn-modified zeolites proved to be more effective in enhancing mortality compared to their non-modified counterparts. Moreover, mixtures of *P. anisum* oil always had greater co-toxicity factors than the respective combinations of *R. officinalis* essential oil, indicating greater synergistic effects.

**Table 10:** Joint action of the essential oils and zeolite mixtures to *C. maculatus* adults.

Binary mixture of essential oils and zeolites (1) + (2)		Calculated mortality (%) at LC_25_ (mg/kg)		Expected mortality (%) (1) + (2)	Observed mortality (%)	Co-toxicity factor
1	2	1	2			
*R. officinalis*	Zeolite-X	24.2	22.5	46.7	56.7	21.4
	Zeolite-A	24.2	20	44.2	55	24.4
	Zn-zeolite-X	24.2	24	48.2	58.3	20.9
	Zn-zeolite-A	24.2	25	49.2	60	21.9
*P. anisum*	Zeolite-X	25	22.5	47.5	60	26.3
	Zeolite-A	25	20	45	58.3	29.5
	Zn-zeolite-X	25	24	49	60.8	24
	Zn-zeolite-A	25	25	50	65	30

Lethal concentration data are means of 4 replicates of 30 insects.

### Effect of zeolite nanoparticles on *C. maculatus* morphology examined by SEM

**Fig. 4:**
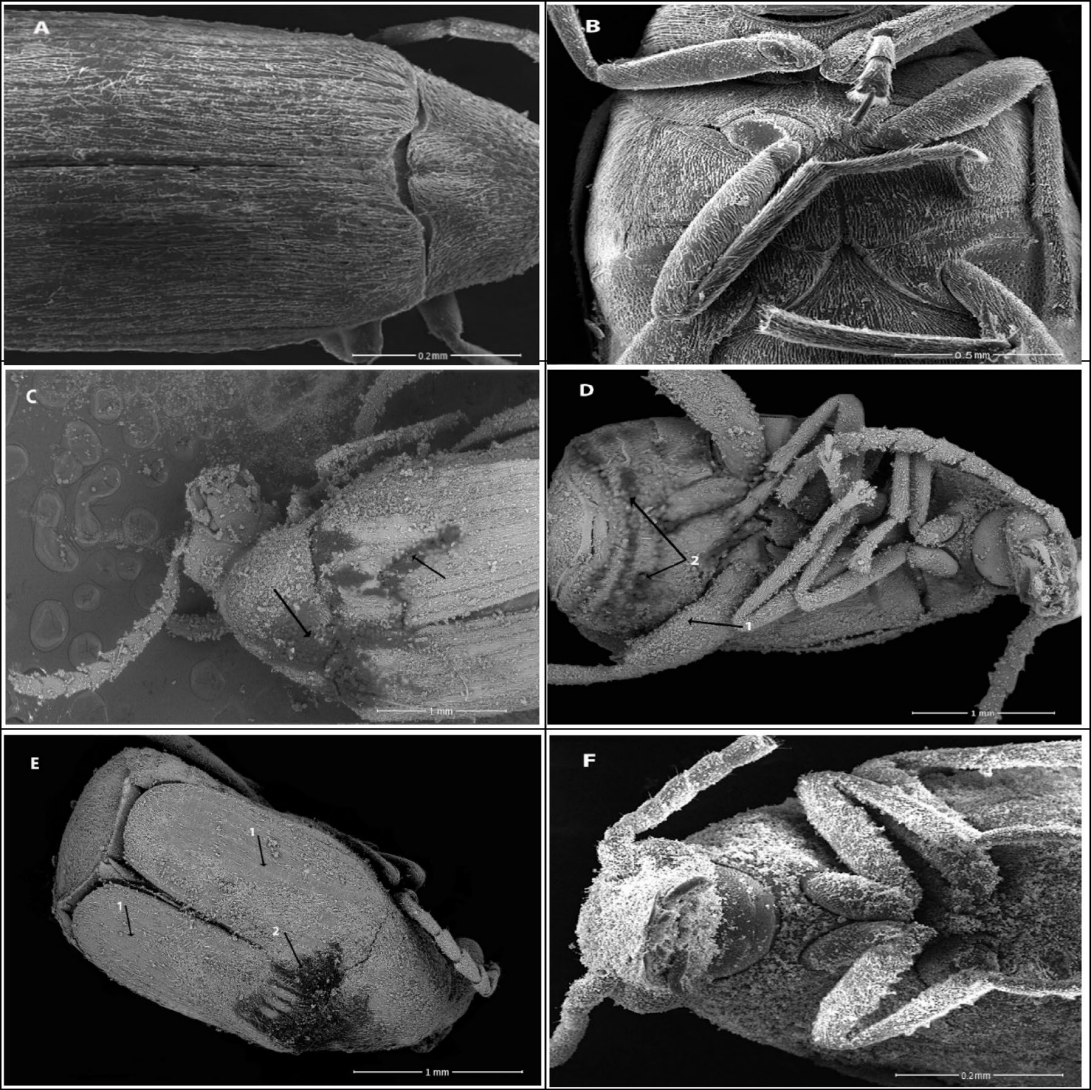
SEM images of *Callosobruchus maculatus* adults. (**A**) and (**B**) Untreated adults’ dorsal and ventral surfaces showing normal cuticle and sensilla shapes. (**C**) The dorsal surface of adults treated with zeolite-A shows desiccation areas (arrows). (**D**) The vertical surface shows the aggregation of zeolite-A particles (arrow 1) and desiccation areas (arrow 2). (**E**) The dorsal surface of adults treated with zeolite-X shows the absence and reduction of the number of sensilla (arrow 1) and desiccation areas on the pronotum (arrow 2). (**F**) Ventral surface showing aggregation of zeolite-X particles on all body surface.

**Fig. 5:**
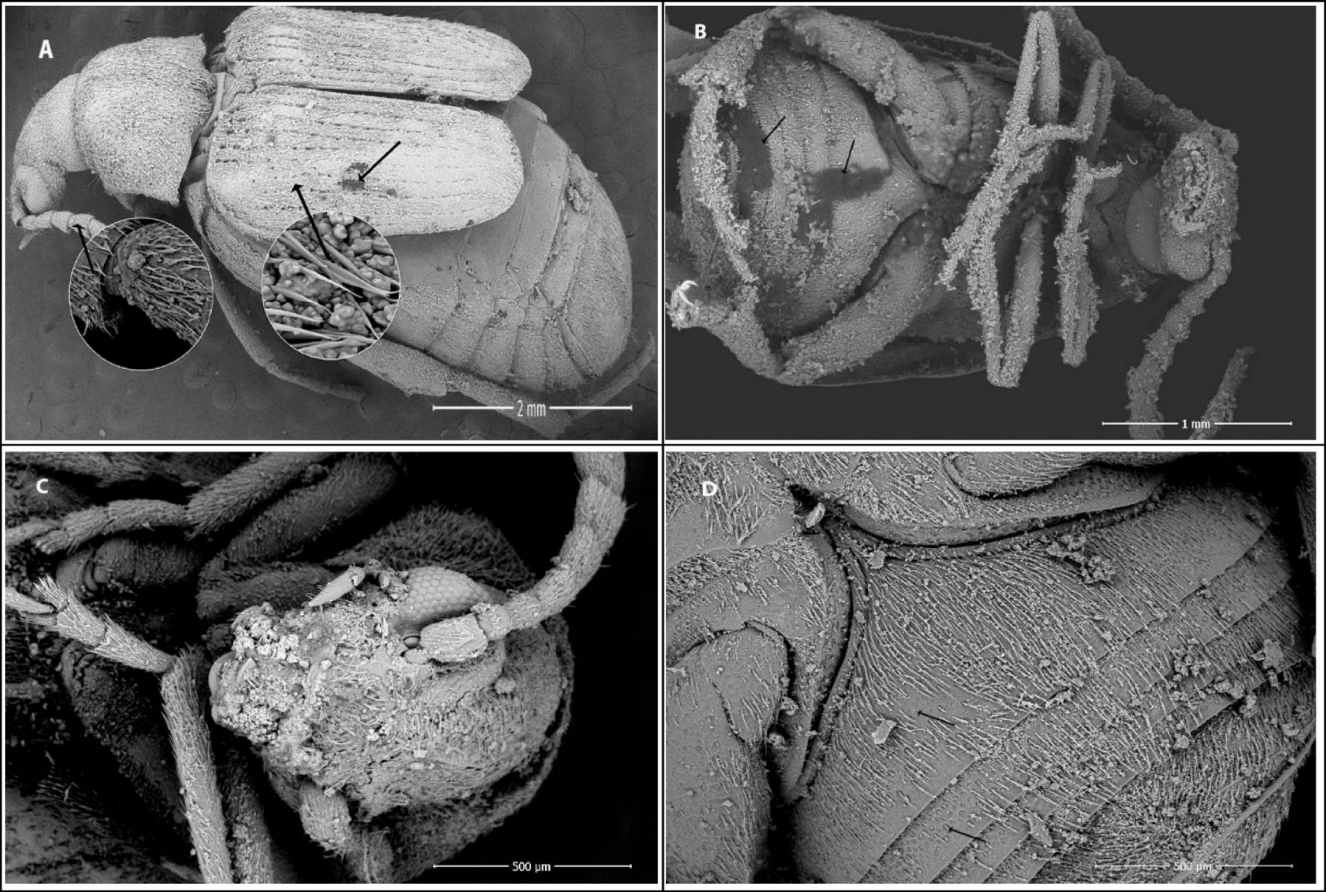
SEM images of *Callosobruchus maculatus* adults. (**A**) The dorsal surface of adults treated with Zn-zeolite-A showed abrasion and distribution of zeolite on the elytra surface and antennae (arrows). (**B**) The ventral surface shows an aggregation of Zn-zeolite-A particles and desiccation areas on the abdomen (arrows). (**C**) The head surface shows an aggregation of Zn-zeolite-X particles. (**D**) The ventral surface of an adult treated with Zn-zeolite-X particles shows the absence and reduction of the number of sensilla (arrows) on the abdomen cuticle.

The SEM images of untreated and treated adults exposed to cowpea seeds treated with zeolite compounds (1000 mg/kg) are shown in Figs. 4 and 5. Zeolite particles revealed a homogeneous distribution of zeolite particles on the cuticle of *C. maculatus* adults and aggregation between the thorax and abdomen joints compared with untreated adults. Image analysis showed that zeolite particles adhered to all body parts. The results also showed that zeolite treatments induced scratches on the elytra and clear damage in sensilla scatters in some points and absent in others, leaving spaces between these parts in the ventral surface, compared with the normal cuticle shape in untreated beetles of *C. maculatus.* Zeolite treatments revealed scratches and splits on the cuticle, leading to water loss through dehydration as the water barrier was damaged and died out of desiccation.

## Discussion

The essential oils of *R. officinalis* and *P. anisum* had different chemical profiles, with *R. officinalis* being rich in oxygenated monoterpenes such as 1,8-cineole, α-pinene, and camphor and *P. anisum* being dominated by E-anethole, an oxygenated monoterpene with a phenylpropanoid structure. These compounds have been reported as insecticidal, repellent, antifeedant, or oviposition deterrent properties against various insect pests^30,31^. Previous studies showed that 1,8-cineole demonstrated considerable contact and fumigant toxicity against various insects, like *Sitophilus* spp, *Tribolium castaneum*,* Rhyzopertha dominica*^32^. Additionally, α-pinene has been found to be more effective than certain commercial products like Detech^®^ (a diatomaceous earth product) against various beetle species^33^. Wang, et al.^34^ stated that trans-anethole had insecticidal efficacy and repellent properties against major stored grain pests.

Zeolites are microporous crystalline aluminosilicates stemming from the reaction of volcanic rocks, ash strata, and alkaline underground water^19,35^. Natural zeolites (alkaline aluminum silicates), depending on their physical characteristics, are the most comparable to diatomaceous earth. Therefore, they can be categorized in the same group as dusts, which contain natural silicates^36^. The synthetic zeolites prepared from kaolin using the microwave technique had high crystallinity and purity, as confirmed by XRD and SEM analyses. The uniformity of zeolite-A in the present study is due to the microwave synthesis, which resulted in homogeneous heating of all reagents at the same time and produced a notable grain-sized regularity^37^. The Si/Al ratios of the synthesized zeolites were consistent with the literature values for zeolite-A and zeolite-X^38^. The cation exchange process successfully doped zeolites with zinc, creating clear crystal faces and edges. This could be due to the corrosive action of acidic Zn-solution on any amorphous relics of metakaolin during the cation exchange process of replacing Na^+^ with Zn^+ 2^. The Si/Al atomic ratio data for zeolite-A and zeolite-X concurred with Youssef, et al.^39^. The diminished amounts of Na^+^ in both samples have been compensated by the high presence of zinc cations. This can indicate a successful and nearly complete replacement procedure between zeolites’ sodium and the cations of Zn^+ 2^ in the immersed solution. The preservation of the crystalline identity of each zeolite type is evidence of the high stability of the framework structure of zeolite-A and zeolite-X under the acidic conditions of the cation exchange process. The zinc loading enhances the catalytic activity of zeolite, improves the porosity, and increases the surface area^40,41^. Zeolitization processing of natural resources usually produces microporous materials containing some relics of the originally treated raw materials^37^. The advantages of using these natural products are their low toxicity to humans and the environment, their biodegradability, and their potential to reduce insect resistance^42,43^.

The biological efficiency of zeolite on stored products has been articulated by many authors^6,19,44^. Zeolite could be used in both open fields and storage facilities^45^. The current research revealed that the mortality of *C. maculatus* adults exposed to zeolite-treated cowpeas increased with increasing concentration and exposure time^6^. Moreover, all the tested zeolite nanoparticles had detrimental effects on the mortality of the tested insect. Zeolites alone or loaded with zinc had moderate insecticidal activity against *C. maculatus* adults. Zeolite-X was more effective than zeolite-A. Data from SEM showed that zeolite-X contained more silica than zeolite-A. Moreover, it possessed a smaller average particle size compared to the larger crystals of Zeolite-A. This implies that the greater efficacy of Zeolite-X can be explained not only by its chemical composition but also by its physical morphology. The smaller particle size gives a higher surface area-to-volume ratio, which probably increases its capacity to abrade the insect cuticle and adsorb epicuticular lipids, thus hastening the lethal process of desiccation. Our findings are consistent with Julbe and Drobek^46^who reported that zeolite-A has a lower Si/Al ratio than zeolite-X, which means it has more cation exchange sites and higher exchange capacities. On the other hand, data from XRD demonstrated that zeolite-X contained minor amounts of zeolite nanoparticles, which means lower Zn substitution than zeolite-A. These results emphasize the mortality results for the insect, as Zn-zeolite-A was more efficient than Zn-zeolite-X. The current research elucidated that loading zinc into zeolites enhanced the insecticidal activity against *C. maculatus.* Zinc loaded on the zeolite surface could exchange with ions in the insect’s body, disrupting the insect’s ionic balance and interfering with their metabolic processes^47^.

The results showed that zeolites and essential oils’ combinations increased the mortality of *C. maculatus* adults and progeny production. The results also clarified that the simultaneous application of essential oils and zeolites significantly reduced the required application rate of zeolites. Korunic and Fields^48^ found that diatomaceous earth and *Anethum graveolens* L. essential oil combination was more effective at lower concentrations against various stored product insects than diatomaceous earth applied alone. At the same time, these lower concentrations of inert dust caused a much smaller reduction in bulk density than the diatomaceous earth used alone. Our results showed that loading essential oils to zeolite exhibited a longer duration of activity and suppressed egg emergence compared to the use of zeolite alone. The combination of essential oils and mesoporous silicates has been recently introduced to enhance the persistence and toxicity of pest management. For example, Ebadollahi, et al.^49^ examined the effect of loading *Eucalyptus largiflorens* essential oil into zeolite as an encapsulated formulation. It was found that the persistence increased from 6 to 17 days for pure and capsulated essential oils, respectively. The current research demonstrated that *P. anisum* essential oil was more efficient to enhance zeolite efficiency than *R. officinalis* essential oil. It might be related to the difference in their chemical constituents, where *P. anisum* essential oil has a higher percentage of oxygenated monoterpenes than *R. officinalis* essential oil. Moreover, *P. anisum* essential oil contained high amounts of E-anethole and methyl chavicol. Wang, et al.^34^ mentioned that *trans*-anethole recorded 100% mortality against the rusty grain beetle adults *Cryptolestes ferrugineus* (Stephens) at 30 mL/L. The effectiveness of traditional synthetic insecticides in the control of *C. maculatus* was emphasized in several studies. These insecticides are usually limited by the development of resistance and environmental hazards. A commercial formulation of β-cyfluthrin had an EC_50_ of 0.51 mg/L against *C. maculatus* on the first day of application^50^. Spinosad dust at 0.3 g/kg (300 mg/kg) reduced progeny by up to 94%^51^. In another study, fumigant Phostoxin was observed to have 86.7% mortality after 48 h at 0.20 mg/kg^52^.

The findings of the current research indicate that binary formulations that include essential oils and zeolites, especially *P. anisum* and Zn-zeolite-A, provide potential directions in biopesticide management of *C. maculatus*. Such synergistic effects are probably multifactorial. Zeolites can act as sustained-release carriers, thereby extending the exposure to volatile compounds. They may trap components of essential oils in their microporous structure to create microenvironments of high concentration that enhance contact toxicity. At the same time, zeolite structures can preserve the volatiles against degradation or evaporation, thus maintaining biological activity during long periods. Milićević, et al.^53^ encapsulated clove essential oil using different types of zeolites to create eco-friendly biopesticides. The formulations showed prolonged efficacy and strong biological activity against various pests and pathogens.

The morphological changes in *C. maculatus* adults exposed to cowpea seeds treated with zeolites showed that zeolites caused scratches, splits, damage, or loss of sensilla on the cuticle, elytra, or ventral surface of *C. maculatus* adults. These changes could impair the cuticle’s protective function and lead to insect dehydration or desiccation. Abdelgaleil, et al.^54^ mentioned that treatment of *C. maculatus* adults with inert dust of diatomaceous earth and kaolin induced general damage to the insect body, like scratches on the elytra and sensilla and traces in scattered areas of the cuticle. Similarly, the SEM results of *S. oryzae* adults treated with zeolite particles showed that zeolite particles adhered to all body parts, including the head, thorax, abdomen, elytra, and legs^55^.

In conclusion, our findings indicated that synthetic zeolites generated from kaolin under microwave conditions had high crystallinity and stability and increased cation exchange capacity after zinc loading. *C. maculatus* mortality rose as zeolite and essential oil concentrations increased. Zeolite-X was more potent than zeolite-A against *C. maculatus*, while loading zinc into zeolites resulted in Zn-zeolite-A being more efficient than Zn-zeolite-X. Combining *P. anisum* essential oil with zeolite caused higher potency against the mortality and progeny production of *C. maculatus* compared to zeolite alone or zeolite and *R. officinalis* combinations at the same concentrations and time durations. Elytra scratches and severe damage to the sensilla spreading of *C. maculatus* were also caused by zeolite treatments. Using zeolite nanoparticles loaded with zinc and combined with essential oils can control *C. maculatus* in stored cowpea seeds. The practical value of these zeolite-based insecticides warrants further research before large-scale implementation.

## Data Availability

The datasets used and analyzed during the current study are available from the corresponding author on request.
